# Isolating Crucial Steps in Induction of Infective Endocarditis With Preclinical Modeling of Host Pathogen Interaction

**DOI:** 10.3389/fmicb.2020.01325

**Published:** 2020-06-18

**Authors:** Christian Schwarz, Verena Hoerr, Yasemin Töre, Vanessa Hösker, Uwe Hansen, Hélène Van de Vyver, Silke Niemann, Michael T. Kuhlmann, Astrid Jeibmann, Moritz Wildgruber, Cornelius Faber

**Affiliations:** ^1^Translational Research Imaging Center, Department of Clinical Radiology, University Hospital Muenster, Muenster, Germany; ^2^Institute of Medical Microbiology, University Hospital Jena, Jena, Germany; ^3^Institute of Musculoskeletal Medicine, University Hospital Muenster, Muenster, Germany; ^4^Institute of Medical Microbiology, University Hospital Muenster, Muenster, Germany; ^5^European Institute for Molecular Imaging, University of Muenster, Muenster, Germany; ^6^Institute for Neuropathology, University Hospital Muenster, Muenster, Germany; ^7^Klinik und Poliklinik für Radiologie, Klinikum der Universität München, Munich, Germany

**Keywords:** endocarditis, foreign body, MRI, *Staphylococcus aureus*, mouse model

## Abstract

Animal models of *Staphylococcus aureus* infective endocarditis (IE), especially in rodents, are commonly used to investigate the underlying pathogenesis, disease progression, potential diagnostic approaches, and therapeutic treatment. All these models are based on surgical interventions, and imply valve trauma by placing a polyurethane catheter at the aortic root. While the influence of endothelial damage and inflammation on the induction of IE has been studied intensively, the role of the catheter, as permanent source of bacteremia, and the interplay with bacterial virulence factors during the formation of IE is poorly understood. In our study, we aimed at identifying which set of preconditions is required for induction and formation of IE: (1) tissue injury, (2) permanent presence of bacteria, and (3) presence of the full bacterial repertoire of adhesion proteins. We investigated the manifestation of the disease in different modifications of the animal model, considering different degrees of endothelial damage and the presence or absence of the catheter. In four infection models the induction of IE was assessed by using two bacterial strains with different expression patterns of virulence factors – *S. aureus* 6850 and Newman. *In vivo* magnetic resonance imaging showed conspicuous morphological structures on the aortic valves, when an endothelial damage and a continuous bacterial source were present simultaneously. Cellular and inflammatory pathophysiology were characterized additionally by histology, real-time quantitative polymerase chain reaction analysis, and bacterial counts, revealing strain-specific pathogenesis and manifestation of IE, crucially influenced by bacterial adherence and toxicity. The severity of IE was dependent on the degree of endothelial irritation. However, even severe endothelial damage in the absence of a permanent bacterial source resulted in reduced valve infection. The spread of bacteria to other organs was also dependent on the pathogenic profile of the infectious agent.

## Introduction

Over the last years the incidence of *Staphylococcus aureus* (*S. aureus*) infective endocarditis (IE) has continuously increased (Bussani et al., 2019). Along with medical progress, intensive treatment conditions such as renal hemodialysis, immunosuppression, and long term indwelling central venous catheters, but also the application of modern cardiac device implants and valve prostheses constitute increasing sources of infection (Hoerr et al., 2018). *S. aureus* is one of the leading pathogens, as it adheres easily through its plethora of adhesins on the surface of implants and is able to form thick multilayered biofilms (Manandhar et al., 2018).

Diagnosis of IE is based on the four columns: clinical symptoms, laboratory parameters, imaging, and microbiology which mainly follow the major and the minor modified Duke criteria (Baddour et al., 2005). Especially with respect to imaging IE, echocardiography remains the gold standard (Habib et al., 2015). In addition to image-based diagnostics, rapid detection and identification of the causative pathogen is decisive for a timely and targeted therapy. Notably, despite correct puncture of a peripheral vein after sufficient disinfection, in up to 35% of the cases blood cultures remain negative and delay the diagnosis (Subedi et al., 2017). As a consequence of delayed diagnosis, complications such as cardiac failure, systemic embolism, abscesses, bleeding, and organ infarction lead to a mortality rate of 30% in patients with *S. aureus* IE (Habib et al., 2015). The formation of *S. aureus* IE implies a series of different host and pathogen factors and can be induced through different pathogenic pathways (Werdan et al., 2014). Frequently the bacterial infestation of the valve is mediated by the two fundamental physiological conditions: endothelial damage of the cardiac valves and circulating bacteria (Angrist, 1963; Werdan et al., 2014). Mechanical trauma of the endothelium results in a rapid fibrinogen-fibrin network with platelet deposition which represents an ideal condition for *S. aureus* to adhere to the impaired cardiac valve. Subsequent interaction between immune cells, platelets, and bacteria as well as cytokines regulates the further growth and development of cardiac valve vegetations (Bancsi et al., 1996; Kobayashi et al., 2015).

To further advance therapeutic and preventive regimens and measures, different animal models of IE have been proposed in the past based on dogs (Highman et al., 1956), horses (Else and Holmes, 1972), pigs (Jones, 1969; Jones, 1982; Johnson et al., 1986), opossums (Rowlands et al., 1970), rabbits (Durack and Beeson, 1972; Durack et al., 1973), and rodents such as rats (Héraïef et al., 1982; Baddour et al., 1984; Gupta et al., 2013; Li et al., 2016) and mice (Gibson et al., 2007; Ring et al., 2014; Liesenborghs et al., 2019). All models depend on similar interventions. In the first instance a mechanical irritation and damage of the endothelium of the cardiac valves is induced by a polyethylene catheter, followed by a hematogenous *S. aureus* infection, providing circulating bacteria which have the capability to adhere to the harmed tissue (Moreillon et al., 2002). In most of the animal studies the catheter remains in place on the cardiac valves during progression of IE until the animal is sacrificed. The presence and underlying role of the catheter during the infection is, however, controversially discussed in pertinent literature, as the catheter representing foreign material rather mimics the infection of a valve prostheses than of naïve valves (Sande and Zak, 1999).

Thus, within the frame of our study we systematically investigated the influence of the degree of valvular tissue damage, the presence of foreign material, as well as the different virulence profiles on the induction of *S. aureus* IE. In four different mouse models of IE with different modifications of valvular damage and catheter placement, two different *S. aureus* infections were examined using the strains 6850 and Newman. Magnetic resonance imaging (MRI) was used to depict the manifestations of bacterial vegetation on the valves, histology to confirm tissue damage and bacterial presence, colony forming units (CFU) counts to quantify bacterial distribution and burden, and clinical scores to represent the overall disease severity.

## Materials and Methods

### Mouse Model of *S. aureus*-Induced Infective Endocarditis

Female C57BL/6 mice (*n* = 144) with a body weight of 16–25 g (average weight: 21.5 ± 1.7 g) and an age of 8–12 weeks were used in this study.

**FIGURE 1:**
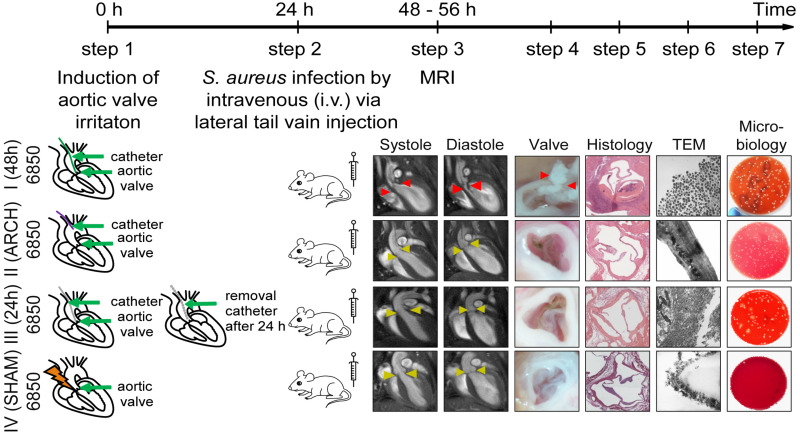
Schematic representation of four different models of *S. aureus* IE. The experimental procedure includes the following steps: First, a 32G-catheter is placed via the right carotid artery on the aortic valves to irritate endothelium (Model I, III, IV) or at the aortic arch to avoid the irritation of the heart valves (Model II) (step 1). *S. aureus* bacteria 6850 or Newman (10^5^ CFU) are intravenous (i. v.) injected 24 h post-surgery through the lateral tail vein (step 2). *In vivo* MRI is performed 48 h after the first surgery (step 3) and animals sacrificed subsequently. The aortic valves are removed, macroscopically investigated (step 4) and prepared for histology (step 5), transmission electron microscopy (TEM, step 6), or microbial analysis (step 7).

For the induction of *S. aureus* IE, a surgical intervention was performed prior to the infection as described previously (Ring et al., 2014). A 32-G polyurethane catheter (13 mm of the tube were cut and heat-sealed at both ends) was placed via the right carotid artery at the aortic root to irritate the aortic valve. The aortic valves were reliably reached by slowly advancing the catheter until the vibration was induced by the fast moving aortic valves. An inoculation with *S. aureus* 6850 or Newman was performed 24 h post catheter placement by intravenous (i. v.) injection through the tail vein (see Figure 1).

Our study included 13 groups of animals comprising a naïve control group and four different surgical models that were each injected via the tail vein with either *S. aureus* 6850, *S. aureus* Newman, or phosphate-buffered saline (PBS; MOCK groups) 24 h after surgical intervention. The four interventions differed in site and duration of catheter placement (see Figure 1):

• Model I: The catheter was placed directly on the aortic valves for 48 h and was present during the whole time of infection. This model combined permanent valve damage with permanent bacteremia due to adherent bacteria on the catheter during progression of IE.
• Model II: The catheter was placed for 48 h in the aortic arch without damaging the aortic valve but was present during the whole time of infection, representing a bacterial source and thus permanent bacteremia. To put the catheter in the aortic arch, the catheter was cut to a length of 1 cm to avoid irritation of the aortic valves.
• Model III: The aortic valves were irritated for 24 h by the catheter to stimulate pre-damage of the heart valve. After the stimulus and prior to infection, the catheter was removed during a second surgical intervention.
• Model IV: A ten-second irritation of the aortic valve was performed with a catheter (SHAM group), assuming that no sustained damages (i.e., not anymore present at the time of infection) occurred at the valves. This model was used to assess the burden of surgery and systemic infection without IE formation.

During surgery, the animals were anesthetized with 2% isoflurane and received medication for peri- and postoperative pain (Carprofen; Rimadyl, Pfizer Animal Health, NY, United States). The infectious dose of 10^5^ CFU was used, as it was previously shown to reliably induce IE (Ring et al., 2014). On the third day of the experiment (48–56 h after catheter placement), the animals were sacrificed by transcardial perfusion either after the last MRI scan as described below or at defined endpoints (clinical score >20, see Supplementary Table S1). Organs and catheter were harvested for microbiological analysis.

### Clinical Score

All animals were scored and examined daily for health status (body weight, body temperature, respiration, physical appearance, behavior, and wound healing from surgical interventions) defined by the approved animal use protocol. Each day, the corresponding score points for each health feature were added up and summarized as clinical score, representing a quantitative measure of the severity of the disease. Design of the clinical score was based on previous studies (Ring et al., 2014; Shrum et al., 2014) and typical IE symptoms (Kreitmann et al., 2020).

### Bacterial Strains

For all infections either the methicillin-susceptible wildtype *S. aureus* strain 6850 (Vann and Proctor, 1987; Fraunholz et al., 2013) or Newman (Duthie and Lorenz, 1952; Baba et al., 2008) was used. Both strains are human isolates. Newman has been used extensively in animal models of staphylococcal disease due to its robust virulence phenotypes (Baba et al., 2008). Supplementary Figure S1 provides a summary of the different pathogenicity profiles of the two strains, which confirmed previous literature. While for *S. aureus* 6850 strong expression (*p* < 0.05) of cytotoxic and hemolytic toxins such as α-toxin (hla) could be demonstrated (Balwit et al., 1994; Haslinger-Loffler et al., 2005; Haslinger-Loffler et al., 2006; Lam et al., 2010; Strobel et al., 2016) the *S. aureus* strain Newman showed low toxin expression (Dassy et al., 1993; Sause et al., 2017), but pronounced adhesin expression such as staphylococcal protein A (SpA; Hartleib et al., 2000) extracellular adherence protein (Eap; Chavakis et al., 2002; Johnson et al., 2008; Mainiero et al., 2010; Hoen and Duval, 2013) Clumping factor A (ClfA; McDevitt and Foster, 1995; Juuti et al., 2004), and Fibronectin binding protein A (FnBPA), which promote the adherence to damaged endothelium at the aortic valves. Bacteria were grown in brain heart infusion (BHI) medium overnight, followed by a 3 h culture after a 1:100 dilution of overnight culture in fresh BHI. The bacteria were washed in PBS, adjusted to OD_578_ = 1 (optical density at 578 nm) and stored at −80°C until use. Colony forming units were enumerated after serial dilution and overnight growth at 37°C on agar plates. Antibiotic-sensitivity testing was performed on fresh *S. aureus* isolates (Automated VITEK^®^ 2 System Version 9.02 (BioMérieux Inc.) (Supplementary Table S2).

### Extraction of Bacterial RNA and RT-qPCR

Extraction of bacterial RNA and real-time quantitative polymerase chain reaction (RT-qPCR), including complementary DNA synthesis and real-time amplification was performed as described previously (Tuchscherr et al., 2011). In order to analyze the expression of the virulence factors α toxin (*hla*), staphylococcal protein A (*spA*), extracellular adherence protein (e*ap*), Clumping factor A (*clfA*) and Fibronectin binding protein A (*fnbA*), the gene for gyrase B (*gyrB*) was taken as the housekeeping gene. All data were normalized to this gene. Primers used in this study are listed in Table 1.

**TABLE 1:** Primers used to study bacterial gene expression.

Gene	Primer	Primer-sequence	References
*gyrB*	Forward	5′-AATTGAAGCAGGCTATGTGT-3′	Tuchscherr et al., 2011
	Reverse	5′-ATAGACCATTTTGGTGTTGG-3′	
*hla*	Forward	5′-CAACTGATAAAAAAGTAGGCTGGAAAGTGAt-3′	Tuchscherr et al., 2011
	Reverse	5′-CTGGTGAAAACCCTGAAGATAATAGAG-3′	
*clfA*	Forward	5′-GAATCAGCTCCACAGAGTACAG-3′	Kalinka et al., 2014
	Reverse	5′-TCTCATTCTAGGCGCACTTG-3′	
*eap*	Forward	5′-AGTCATTGATTACAACAA-3′	Joost et al., 2009; Kalinka et al., 2014
	Reverse	5′-CTTATTAAATGTTAAGCTTG-3′	
*fnbA*	Forward	5′-ACAAGTTGAAGTGGCACAGCC-3′	Tuchscherr et al., 2011
	Reverse	5′-CCGCTACATCTGCTGATCTTGTC-3′	
*spA*	Forward	5′-CAGATAACAAATTAGCTGATAAAAACAT-3′	Roberts et al., 2006; Kalinka et al., 2014
	Reverse	5′-CTAAGGCTAATGATAATCCACCAAATAC-3′	

### MR Imaging

Measurements were performed at 9.4T on a BioSpec 94/20 small animal MRI system (Bruker BioSpin, Ettlingen, Germany) equipped with a 1 T/m gradient insert (BGS-6S, Bruker) and ParaVision software version 5.1 (Bruker). A mouse body quadrature volume coil with an inner diameter of 35 mm (Rapid Biomedical, Rimpar, Germany) was used for image acquisition. During MRI, the mice were anesthetized with isoflurane (1.5–2.5% isoflurane and 0.7/0.3 air/O_2_ mixture), and core body temperature and respiration rates were monitored using a MRI compatible monitoring system (SA Instruments, Stony Brook, NY, United States). A self-gated CINE-UTE sequence was used to visualize the infected aortic valves over the cardiac cycle (TR/TE: 5/0.31 ms, FA: 15°, FOV: (3.20 cm^2^), MTX: 256 × 256, section: 1 mm, scan duration: 12:08 min). 20 images per cardiac cycle were reconstructed retrospectively.

### *Ex vivo*-Analysis

#### Microbiological Analysis

Spleen, lung, kidney, liver, myocardium, aortic arch, and aortic valves were homogenized, serially diluted in PBS and plated on blood agar plates for bacterial counting. The isolated catheter was first placed in an ultrasound bath for 10 min and then vortexed for 1 min. The catheter supernatant was serially diluted and spread on blood agar plates. All blood agar plates were incubated overnight at 37°C and CFUs were counted subsequently. Corresponding CFUs were determined per mg tissue or catheter, and mean values were plotted in a spider chart. For each chart the area under the curve [AUC, shoelace formula (Braden, 1986)] was calculated, representing the overall bacterial burden of each animal group investigated.

### Histology

Serial sections of paraffin-embedded heart valves were prepared with a thickness of 2, 5 or 10 μm for histological analysis. The gram-positive *S. aureus* bacteria were stained with crystal violet/iodine (GRAM) according to standard protocols. Hematoxylin and eosin staining (HE) was used to detect basophilic components (DNA/RNA) and eosinophilic structures (collagen/muscle), and Weigert’s Elastica-van-Gieson (EvG) staining was used to label collagen to indicate changes in the blood vessel (Kazlouskaya et al., 2013). Tissue sections were examined under the light microscope (Nikon Eclipse 50i, Nikon-Düsseldorf, Germany) at 60x and 100x primary magnification.

### Transmission Electron Microscopy

Aortic valves were removed from each experimental group 24 h post infection and analyzed by transmission electron microscopy (TEM). In brief, heart valves or catheters were fixed in 2% (v/v) formaldehyde and 2.5% (v/v) glutaraldehyde in 100 mM sodium cacodylate buffer, pH 7.4, at 4°C overnight. For heart valves: After washing in PBS, samples were postfixed in 0.5% (v/v) osmium tetroxide and 1% (w/v) potassium hexacyanoferrate III in 100 mM sodium cacodylate buffer for 2 h at 4°C followed by washing with distilled water. After dehydration in an ascending ethanol series from 30 to 100% ethanol, samples were two times incubated in propylenoxide each for 15 min and embedded in Epon (Epoxy Embedding Medium Kit; Sigma, Taufkirchen, Germany) using a flat embedding mold. For catheters: After dehydration in ethanol up to 70%, samples were embedded in LR White medium (London Resin Company, London, United Kingdom), and polymerized using UV light according to the manufactures instructions. Ultrathin sections were cut with an ultramicrotome (Leica Ultracut UCT), collected on copper grids and negatively stained with 2% uranyl acetate for 20 min. Electron micrographs were taken at 60 kV with a Phillips EM-410 electron microscope using imaging plates (Ditabis, Pforzheim, Germany).

### Immunohistology

The 2 μm thick sections of the heart valves were dewaxed and boiled in citrate buffer (pH 6) (2 × 2 min). Subsequently, the primary antibody von-Willebrand-factor (vWF) (ab6994; Abcam, United Kingdom; dilution 1:200) was added and incubated overnight at 4°C. Next, unbound primary antibody vWF was removed from the slice and secondary antibody goat-anti-rabbit (Biotin) (B2770 life technologies dilution 1:600) was added to the sections and incubated at room temperature for 1 h. Subsequently the label Alexa Fluor 546 with Streptavidin-Tag (dilution 1:800) was incubated for 45 min at room temperature to bind to the biotin. Finally, DAPI staining, incubating slices for 6 min at room temperature, was used to display the cell nuclei.

### Statistical Analysis

Statistical analyzes were performed with the software GraphPad software version 5 (GraphPad Software, La Jolla California United States). For each IE model (I–IV) bacterial titers were analyzed statistically using a one-way ANOVA calculation. Bacterial load on the valves was compared to that in all other organs after Bonferroni correction. A value of *p* < 0.05 was considered as significant.

### Ethics Statement

All animal experiments were approved by the North Rhine-Westphalia Agency for Nature, Environment, and Consumer Protection (Landesamt für Natur, Umwelt und Verbraucherschutz Nordrhein-Westfalen-LANUV; ID 87-51.04.2011.A003; ID 84-02.04.2015.A581).

## Results

### Magnetic Resonance Imaging

**FIGURE 2:**
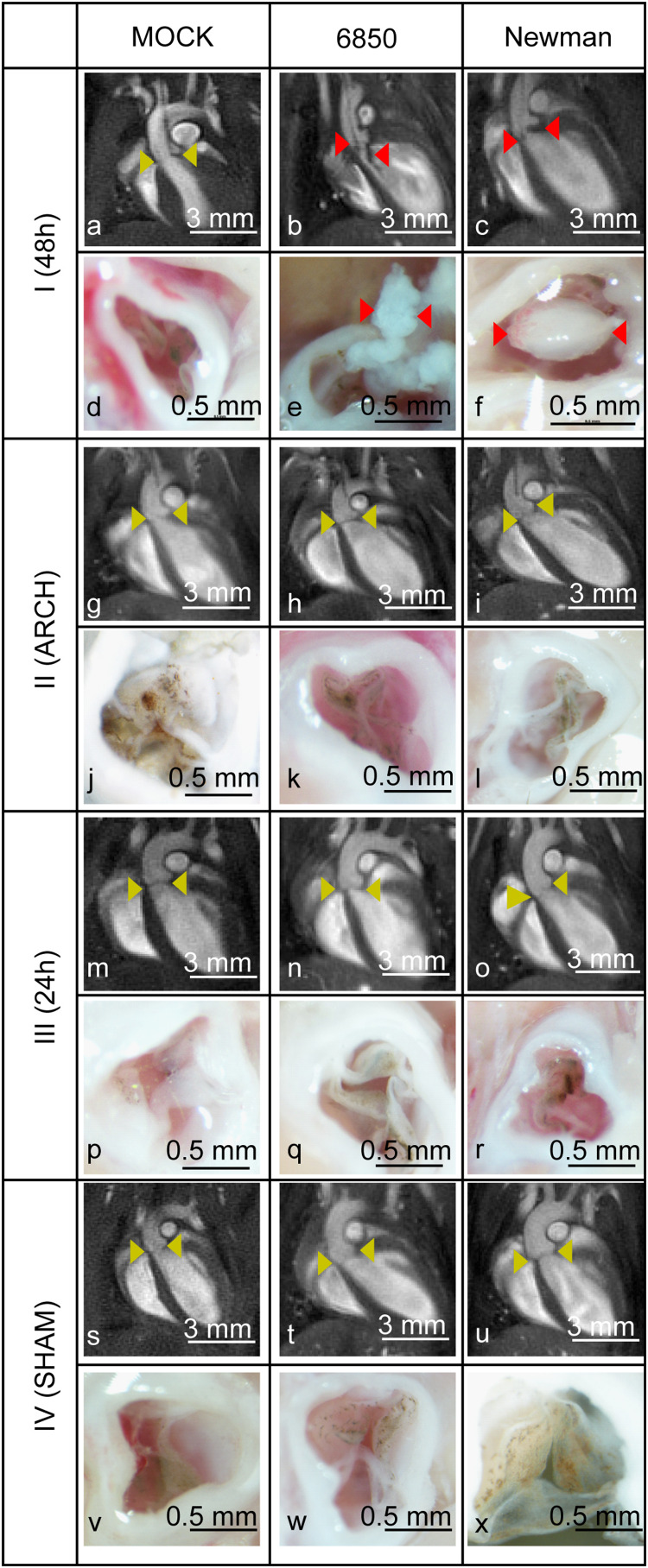
MR- (upper row) and macroscopic- (lower row) images of all four models of IE (I-IV) using *S. aureus* 6850, Newman and PBS as control (MOCK group). Aortic valves without abnormalities **(a,g,h,i,m–o,s–u)** are highlighted by yellow and insufficient aortic valves with bacterial vegetations **(b,c,e,f)** by red arrow heads. The white scale bar in the MR images represents 3 mm. The black bar in the macroscopic images refers to 0.5 mm.

Four mouse models of IE with different surgical interventions (I–IV, Figure 1), were infected with the *S. aureus* strain 6850 or Newman and were examined with respect to abnormal valve formations by *in vivo* MRI (see Supplementary Movies S1–S12). Only the IE model I demonstrated thickened heart valves after inoculation with either *S. aureus* strain (Figure 2; also see Supplementary Movies S2, S3). Especially *S. aureus* 6850-infected valves presented additional structures such as for example pendulum-like masses. On these valves the presence of pronounced vegetations could be confirmed by macroscopic examination after the organ had been harvested. Black-brown deposits could further be identified on the valves of perfused hearts due to the aggregation of thrombocytes (see Figures 2d,j,p,v) during the infection (Liesenborghs et al., 2019). The other three modified models of IE (II–IV) showed fragile and healthy valve structures similar to those of the MOCK groups having received a PBS injection.

### Histology

**FIGURE 3:**
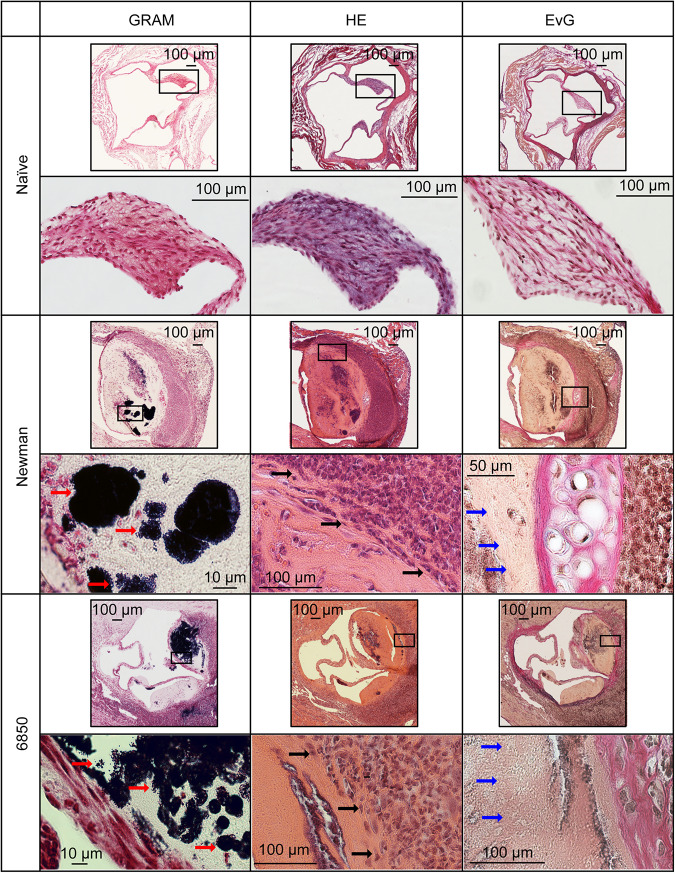
Histology of the aortic valves in IE model (I) 24 h post infection. Representative sections are shown from naïve valves as well as from valves infected with *S. aureus* Newman and 6850. Black rectangles indicate where magnifications were taken Gram staining shows large colonies of Gram-positive *S. aureus* bacteria (red arrows). Hematoxylin-eosin (HE) staining reveals the recruitment of neutrophils (black arrows). Weigert’s Elastica-van-Gieson (EvG) staining labels elastic fibers (black) and collagen (red), illustrating changes in blood vessel walls. *S. aureus* induces development of amorphous structures, which appears yellowish in EVG (blue arrows).

To examine morphological conspicuities of the heart valves such as thickening and additional structures in IE model I, histological analysis was performed on valve sections using GRAM, HE and Weigert’s EvG staining. In naïve control animals, no evidence of inflammation was found in either staining. In contrast, animals with *S. aureus* infection and irritated aortic valves, clearly revealed bacterial vegetations on the aortic valves and also partly in the adjacent soft tissue. The bacteria formed extended clusters on the valves resulting in occasionally solid amorphous structures (Figure 3, red arrows). These structures could not be identified as collagen in Weigert’s EvG staining (blue arrows). Especially at the root of the valve and at the adjacent tissue a fulminant infiltration and accumulation of immune cells, i.e., mainly neutrophilic granulocytes (HE staining, black arrows) were observed with both *S. aureus* strains. Unambiguous strain-specific morphological manifestations in the valvular cellular structures during IE could not be identified.

### Colony Forming Unit

**FIGURE 4:**
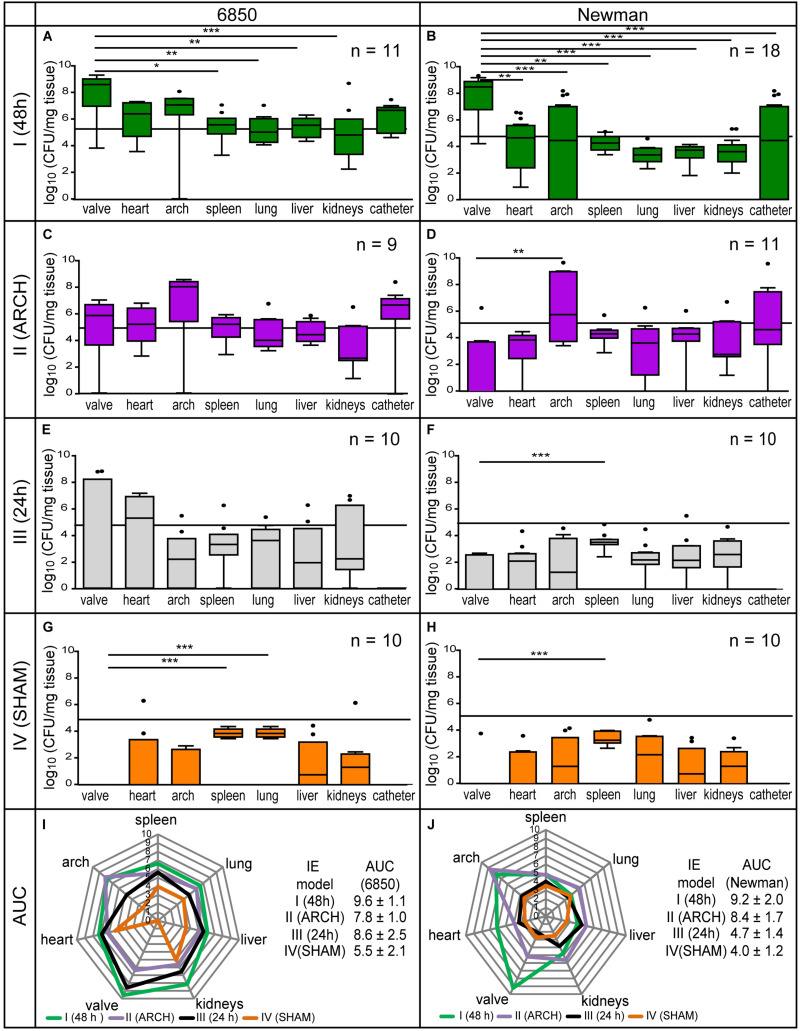
Microbial analysis of four different models of IE (I–IV) using the *S. aureus* strains 6850 and Newman. **(A,B)** Shows model I, **(C,D)** shows model II, **(E,F)** shows model III, **(G,H)** shows model IV. The bacterial injection dose of 10^5^ CFU is marked as a black threshold in the respective graphs of the corresponding bacterial counts in different organs. The results are displayed as box and whiskers plots, with data between first and third quartiles, the band in the box stands for the second quartile (=median). Whiskers represent lowest and highest data within 1.5 interquartile ranges of the lower and upper quartile. The overall bacterial load is shown as spider web chart for all four models of IE induced with *S. aureus* 6850 **(I)** and Newman **(J)**, 24 h post infection. For each model the corresponding bacterial burden is calculated as area under the curve (AUC). *p < 0.05, **p < 0.01, ***p < 0.001.

To assess the extent of localized *S. aureus* vegetations on the aortic valves, bacterial distributions of either *S. aureus* strain (Newman or 6850) were investigated in all four models of IE (I–IV) 24 h post infection by counting CFUs (Figure 4). For both strains, the aortic valves of model I revealed an average bacterial load of 10^9^ CFU / mg tissue and thus differed significantly from the results in spleen, lung, liver, and kidney (Figures 4A,B). Infections with the strain 6850 showed similar bacterial load (AUC = 9.6 ± 1.1, Figure 4I) compared to Newman infections (AUC = 9.2 ± 2.0, Figure 4J).

**FIGURE 5:**
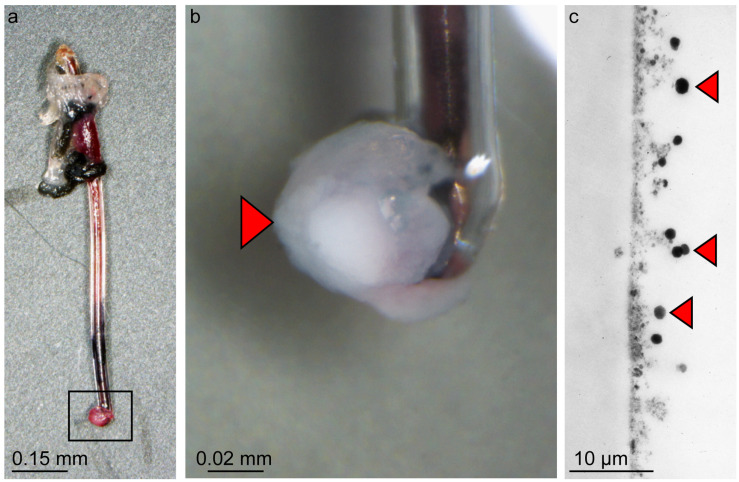
Catheter of model II 24 h post infection showing adherent *S. aureus* Newman bacteria. **(a)** Macroscopic image of a Newman-infected catheter with additional structures at the tip (black rectangle). **(b)** Seven fold magnification of the additional structure (red arrow). **(c)** TEM image of the surface of the catheter with cocci of bacteria seen as large black dots, red arrows, 21.000-fold magnification).

In contrast, in model II, having the catheter placed in the aortic arch instead of the aortic root, the highest bacterial load (6850: 10^8^ CFU/mg tissue, Newman: 10^6^ CFU/mg tissue) was counted in the aortic arch. Local infection was higher than on the aortic valves (6850: 10^6^ CFU/mg tissue, Newman: 10^4^ CFU / mg tissue), representing a significant difference in bacteria counts in Newman infections (Figures 4C,D). Altogether, in model II, Newman infections (AUC = 8.4 ± 1.7, Figure 4J) resulted in a greater over all bacterial burden in comparison to infections induced with *S. aureus* 6850 (AUC = 7.8 ± 1.0, Figure 4I). Macroscopic and microscopic analysis of the catheter revealed adherent bacteria and bacterial vegetations on the catheter tip, providing a permanent source of infection (Figure 5).

In model III, an irritation was performed by the catheter on the aortic valves for 24 h only. In infections with strain 6850, a high bacterial load of 4 × 10^8^ CFU/mg tissue was counted on the heart valves. Bacterial burden was similar in all organs investigated (Figure 4E). In contrast, for Newman infections only low levels of CFU values were counted on the aortic valves as well as in all other organs (up to 10^3^ CFU/mg tissue, Figure 4F). Infections with *S. aureus* 6850 (AUC = 8.6 ± 2.5, Figure 4I) resulted in approximately twice as high over all bacterial burdens as compared to infections with *S. aureus* Newman (AUC = 4.7 ± 1.4, Figure 4J).

In model IV, the shortest irritation time of the aortic valve was used among all four models. Inoculation with either *S. aureus* 6850 or Newman resulted in the overall lowest bacterial burden compared to the other three IE models (I–III). Lung and spleen showed the strongest infection (6850: 10^4^ CFU/mg tissue; Newman: 10^3^ CFU/mg, Figures 4G,H) and no bacteria were found on the heart valves. In general, in model IV infections with *S. aureus* 6850 (AUC = 5.5 ± 2.1, Figure 4I) led to a higher bacterial load than infections with *S. aureus* Newman (AUC = 4.0 ± 1.2, Figure 4J).

**FIGURE 6:**
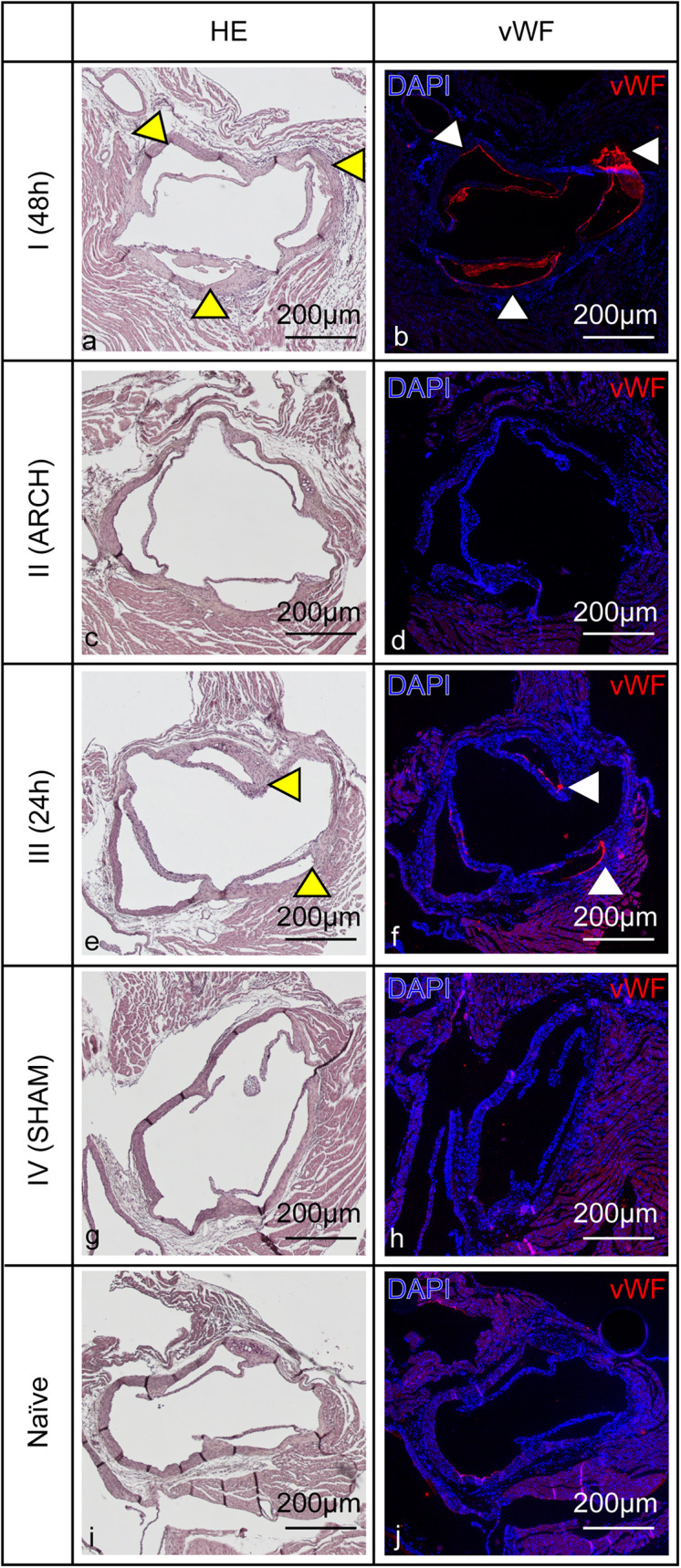
Endothelial damage of the aortic valves induced by corresponding catheter placement in all four models of IE (I–IV) with MOCK-infection. **(a,c,e,g,i)** Hematoxylin-eosin staining shows anatomical cellular structures of the aortic valves. **(b,d,f,h,j)** Cell nuclei are visualized by DAPI fluorescence staining, immunohistological staining identifies vWF expression especially in model I **(b)** and III **(f)** (red signal, white arrow heads). Hematoxylin-eosin staining shows anatomical cellular structures of the aortic valves (left column). Cell nuclei are visualized by DAPI fluorescence staining (blue signal in the right column). Immunohistological staining identifies vWF expression especially in model I and III (red, white arrow heads). Corresponding anatomical structures are pointed out with yellow and white arrow heads. Scale bars indicate 200 μm.

To further assess the underlying degree of endothelial damage, immunohistological investigations were performed in all four models of IE, 24 h post mock infection, by qualitative analysis of vWF expression (Goncharov et al., 2017) on the aortic valves. Those revealed pronounced vWF expression (red) in model I and minor vWF expression in model III (Figure 6). No vWF expression was detected in models II and IV as well as in healthy naïve animals.

Altogether, our results showed that model I involving both endothelial damage as well as a continuous bacterial source such as adherent bacteria on the catheter, lead to the most pronounced manifestation of IE.

### Clinical Score

**FIGURE 7:**
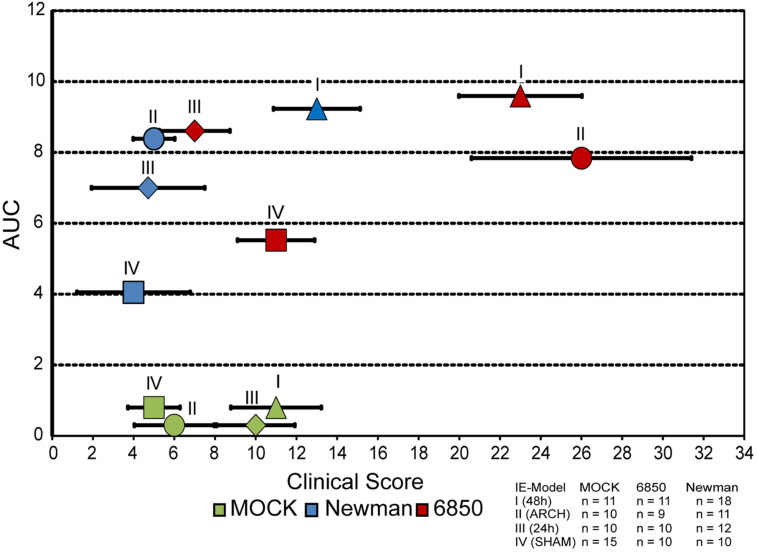
Correlation of bacterial load measured as AUC (see Figure 4) and clinical score, in all four models of IE induced with either *S. aureus* 6850 or Newman. Mean clinical score (±SEM) of each experimental group at 24 h post infection is shown.

All four mouse models of IE were evaluated with regards to their clinical scores, which were then correlated to the corresponding bacterial burden. The MOCK groups of all four different models of IE (I–IV) that did not receive any pathogen infection showed mild to moderate clinical scores in the range of 4–12 (Figure 7). In Newman infections, almost the same clinical scores were observed in the corresponding models of IE, even though higher overall bacterial counts with AUC values up to levels of 10^9^ CFU/mg tissue and 10^8^ CFU/mg tissue were observed in model I and II, respectively. In contrast, inoculation of mice with *S. aureus* 6850 resulted in high clinical scores of 23 ± 3.0 and 26 ± 5.4 in IE model I and II, respectively, while similar bacterial burdens of 10^9^ CFU/mg tissue and 10^8^ CFU/mg tissue were observed, respectively. These animals clearly showed severe symptoms of sepsis such as weight loss, lethargy, as well as a high bacterial load in all organs studied. Model III and IV showed only mild symptoms of infection and also revealed the lowest bacterial counts in spleen, lung, liver, and kidney. These models were scored with similar clinical findings as the corresponding MOCK groups. Taken together, our results reveal a discrepancy between number of bacteria on the valves and clinical symptoms. While the extent of local vegetations on the heart valves mainly depended on degree of tissue injury and presence of bacteria in the blood stream, the clinical picture was dominated by pathogenicity and virulence factor profile of the respective *S. aureus* strain.

## Discussion

In this study, the differential impact of host and pathogen factors on severity of IE was investigated by imaging and microbial analysis of four different surgical IE models, each infected with two different *S. aureus* strains. Several animal models have been established over the last years (Highman et al., 1959; Garrison and Freedman, 1970; Durack and Beeson, 1972; Durack et al., 1973; Baddour et al., 1984; Sande and Zak, 1999; Gibson et al., 2007; Veloso et al., 2011) to advance the understanding of the underlying pathogenesis of IE and to improve current diagnostic and therapeutic interventions. All animal models of IE share comparable procedures, beginning with valve trauma through mechanical irritation by placing a catheter on the aortic root, followed by intravenous infection with a bacterial dose between 10^5^ and 10^8^ CFUs (Gibson et al., 2007; Ring et al., 2014; Liesenborghs et al., 2019). In many models the catheter remains in place during the induction of infection and possibly has a high impact on disease progression due to adherent bacteria on the catheter forming a permanent source of infection (Baddour et al., 1984; Sande and Zak, 1999; Ring et al., 2014). In our study, we systematically investigated the influence of the catheter and endothelial damage on formation of bacterial vegetations on the aortic valves and overall bacterial distribution. To this end, in the models we either placed the catheter at the aortic root for 48 h (I), 24 h (III) or 10 s (IV, SHAM) or at the aortic arch for 48 h (II) and infected the animals with *S. aureus* 6850 or Newman 24 h post-surgery.

### Damage

In consideration of the different bacterial burdens found on the aortic valves in all four models of IE, our results demonstrate that the endothelial damage is one of the key factors in the induction of IE. Only in model I, having a catheter placed at the aortic root for 48 h, pronounced vegetations of *S. aureus* 6850 and Newman could be detected on the valves in MR and macroscopic images. Histology and CFU counts confirmed the presence of large amounts of bacterial colonies in the range of 10^9^ CFU/mg tissue as well as immune cell infiltration. Compared to the models II-IV, the irritation of the aortic valves for 48 h in model I induced severe endothelial activation and injury resulting in strong release of vWF (Sadler, 1998; Starke et al., 2011; Goncharov et al., 2017; Mehta et al., 2019; Figure 6). In model III only mild vWF-expression was detected, while in model IV the irritation of the aortic valves for 10 sec did not result in detectable vWF expression. Severe damage seems to promote strong bacterial adhesion (Liesenborghs et al., 2019). It is well known that vWF, an adhesive glycoprotein which is located in the subendothelial layers of the blood vessel wall, such as the intima layer (Sadler, 1998; De Meyer et al., 2009) is one of the key binding factors during the formation of *S. aureus* IE (Pappelbaum et al., 2013; Claes et al., 2017; Liesenborghs et al., 2018). At sites of endothelial damage and injury, vWF presents on the vascular surface and promotes the adherence of *S. aureus* through its virulence complexes of Clumping factor A (ClfA; Claes et al., 2017) and von Willebrand factor-binding protein (vWbp; Bjerketorp et al., 2004). Both *S. aureus* 6850 and Newman showed comparable numbers of bacteria on the valves.

### Bacteremia

In addition to the strong endothelial damage induced by the catheter in model I, the catheter represented also a permanent source of infection. Besides an intensive inoculation of the heart valves, other organs such as liver, kidney, spleen, lung, heart, and aortic arch also showed high bacterial counts, indicating an unspecific systemic bacterial infection, possibly caused by the bacterial adherence on the catheter (von Eiff et al., 1999; Höök and Foster, 2000; Maira-Litrán et al., 2002; O’Grady et al., 2002; Pant et al., 2018). Model II containing the catheter at the aortic arch without damaging the endothelial layers of the aortic valves also resulted in an overall high number of bacterial counts in different organs. Both with (model I) and without (model II) endothelial damage, the bacterial infection resulted in a similar overall bacterial manifestation and burden, with a high number of bacteria on the aortic valves. These results suggest that the presence of a permanent bacterial source is decisive for the formation of IE. Comparison of model II–IV and I–III support these findings. The presence of the catheter with or without underlying endothelial damage leads to an almost two fold higher overall bacterial burden (AUC values). In previous studies it was shown that the presence of a catheter during the infection enhanced the infective dose (Zimmerli et al., 1982).

### Clinical Picture

Our results suggest that the overall bacterial distribution and the amount of substantially infected organs, rather than the bacterial load on the aortic valve, influence the clinical picture (Singer et al., 2016; Xie et al., 2017). The highest disease severity was found in model I and II with 6850 infections, while all other infection models showed only moderate infection symptoms, similar to animals with MOCK infections (Supplementary Figure S2).

### Adhesion

Even though both in model I and II similar bacterial concentrations were found at the aortic valves for *S. aureus* 6850 and Newman, both the clinical picture and manifestations of the corresponding bacterial vegetation differed substantially on macroscopic levels. *S. aureus* IE induced by the bacterial strain 6850 led to fulminant additional structures on the valves composed of *S. aureus* bacteria, immune cells and connective tissue (Figure 3), in agreement with previous studies (Kolar and Liu, 2016). These structures presented along with severe tissue damage and destruction. In contrast, *S. aureus* Newman infections generally occurred in form of valve thickening. Even though, histologically, no substantial differences in the tissue morphology could be found, possible reasons for these macroscopic observations might be found in the bacteria-specific adhesin and toxin profiles and thus differences in pathogenicity. Both *S. aureus* 6850 and Newman produce fibronectin binding proteins (FnBPs; Supplementary Figure S1). However, in *S. aureus* Newman, despite high expression levels, they are not anchored to the cell wall, which leads to a loss of FnBP-dependent functions (Haslinger-Loffler et al., 2005). In contrast *S. aureus* 6850 expresses low levels of this adhesin on the cellular surface, which mediates efficient adherence to activated and injured tissue (Vann and Proctor, 1987; Balwit et al., 1994; Haslinger-Loffler et al., 2005; Haslinger-Loffler et al., 2006; Lam et al., 2010; Seidl et al., 2011; Seidl and Zinkernagel, 2013; Strobel et al., 2016). In 6850, high expression levels of hemolytic peptides such as α- (Berube and Wardenburg, 2013; Surewaard et al., 2018), β-, γ- and δ- toxins were previously described and verified, which can cause necrotic death of the target host cell and thus induce severe tissue destruction (Vandenesch et al., 2012). There is increasing evidence that the strain is also able to escape from phagolysomes in immune cells such as macrophages, neutrophils and dendritic cells. Through the expression of phenol-soluble modulin and β-toxin (Strobel et al., 2016) the bacteria strain is less vulnerable to the host response system and promotes cell destruction (Giese et al., 2011; Grosz et al., 2014). Even though different adhesin profiles are discussed and observed for 6850 (Balwit et al., 1994) and Newman (Novick, 2003; Traber et al., 2008; Supplementary Figure S1), equal bacterial counts were observed on the catheter for both strains in models I and II (Figures 4A–D). However, in both models *S. aureus* 6850 induced more severe infections of other organs such as lung, liver spleen and kidney, and resulted in higher clinical scores. The aggressive character of the *S. aureus* 6850 became also obvious when comparing the ability of the two *S. aureus* strains to induce IE in model III. The short irritation of the aortic valves for 24 h resulted in decreased vWF release as evidenced by immunohistological analysis. Yet, high bacterial counts on the aortic valves and the heart were observed for infections with 6850, but not for infections induced by *S. aureus* Newman. In contrast to models I and II, in absence of a persisting catheter even *S. aureus* 6850 induced only a moderate bacterial load in the other organs such as liver, spleen, lung and kidney. At the same time, these animals showed milder disease symptoms and reached clinical score values between 4 and 8, which were comparable to those of MOCK infected animals.

## Conclusion

In conclusion, the four different modifications of IE animal models, investigated in our study using two different *S. aureus* strains, take major steps of the pathogenesis of *S. aureus* IE into account – bacterial adhesion at the site of endothelial damage, constant bacteremia and cellular and inflammatory response. Our results showed, that independent of the bacterial strain, pronounced bacterial vegetations on the valves were only formed when the two key factors of endothelial damage and continuous bacterial source were present simultaneously. To induce sufficient endothelial damage, an irritation of the aortic valves by the catheter for at least 24 h was necessary. The longer the catheter remained in place at the aortic valve, the greater the endothelial damage. However, severe endothelial damage in the absence of a permanent bacterial source resulted in reduced valve infection and was strongly dependent on the pathogenic profile of the infectious agent. In contrast, the presence of a foreign body and thus a permanent bacterial source alone caused unspecific overall high bacterial loads and distributions in major organs such as kidney, liver, spleen, lung and heart. The amount of bacteria strongly differed between bacterial strains, which might be regulated by the bacteria-specific affinity to tissue and the catheter. These findings underline that the endothelial activation and injury is the major cause to trigger and promote the formation of IE. The catheter itself additionally fosters the bacterial distribution in other organs and overall systemic infection and its severity.

## Data Availability Statement

All datasets generated for this study are included in the article/Supplementary Material.

## Ethics Statement

The animal study was reviewed and approved by Landesamt für Natur, Umwelt und Verbraucherschutz Nordrhein-Westfalen-LANUV.

## Author Contributions

CF, CS, and VHoe designed the study. AJ (histology), CS (animal models, histology, microbiology, MRI), HV (microbiology), SN (PCR), UH (TEM), VHös (histology, microbiology), VHoe (histology, MRI), and YT (animal models) performed experiments. AJ (histology), CS (animal models, histology, microbiology, MRI), HV (microbiology), MK (histology), SN (PCR, microbiology), UH (TEM), VHös (histology, microbiology), and VHoe (histology, MRI) analyzed data. CF, CS, MW, and VHoe wrote the manuscript. All authors interpreted data, and revised, and edited the manuscript.

## Conflict of Interest

The authors declare that the research was conducted in the absence of any commercial or financial relationships that could be construed as a potential conflict of interest.

## Supplementary Material

FIGURE S1 Gene-expression of the α toxin (hla) and different adhesion molecules such as staphylococcal protein A (spA), extracellular adherence protein (eap), Clumping factor A (clfA) and Fibronectin binding protein A (fnbA) in *S. aureus* 6850 (white boxes) and Newman (gray boxes). The results (*n* = 6 out of three independent cultures) are displayed as box and whiskers plots, with data between first and third quartiles, the band in the box stands for the second quartile (=median). Whiskers represent lowest and highest data within 1.5 interquartile ranges of the lower and upper quartile. ^∗^*p* < 0.05, ^∗∗^*p* < 0.01.

FIGURE S2 Correlation of number of infected organs and clinical score, in all four models of IE induced with either *S. aureus* 6850 or Newman. Mean clinical score (±SEM) of each experimental group at 24 h post infection and the corresponding number of infected organs (≥10^5^ CFU / mg tissue, see Figure 4) is shown.

TABLE S1 Clinical Score.

TABLE S2 Antibiotype of Staphylococcus aureus strains 6850 and Newman.

MOVIE S1 Exemplary CINE MRI for model I (48 h) with PBS injection (AVI).

MOVIE S2 Exemplary CINE MRI for model I (48 h) with *S. aureus* 6850 injection (AVI).

MOVIE S3 Exemplary CINE MRI for model I (48 h) with *S. aureus* Newman injection (AVI).

MOVIE S4 Exemplary CINE MRI for model II (ARCH) with PBS injection (AVI).

MOVIE S5 Exemplary CINE MRI for model II (ARCH) with *S. aureus* 6850 injection (AVI).

MOVIE S6 Exemplary CINE MRI for model II (ARCH) with *S. aureus* Newman injection (AVI).

MOVIE S7 Exemplary CINE MRI for model III (24 h) with PBS injection (AVI).

MOVIE S8 Exemplary CINE MRI for model III (24 h) with *S. aureus* 6850 injection (AVI).

MOVIE S9 Exemplary CINE MRI for model III (24 h) with *S. aureus* Newman injection (AVI).

MOVIE S10 Exemplary CINE MRI for model IV (SHAM) with PBS injection (AVI).

MOVIE S11 Exemplary CINE MRI for model IV (SHAM) with *S. aureus* 6850 injection (AVI).

MOVIE S12 Exemplary CINE MRI for model IV (SHAM) with *S. aureus* Newman injection (AVI).

## References

[B1] Angrist A. A. (1963). Pathogenesis of Bacterial Endocarditis. *JAMA* 183 249–252. 14013194 10.1001/jama.1963.63700040009010b

[B2] Baba T. Bae T. Schneewind O. Takeuchi F. Hiramatsu K. (2008). Genome sequence of *Staphylococcus aureus* strain Newman and comparative analysis of staphylococcal genomes: polymorphism and evolution of two major pathogenicity islands. *J. Bacteriol.* 190 300–310. 10.1128/jb.01000-07 17951380 PMC2223734

[B3] Baddour L. M. Christensen G. D. Hester M. G. Bisno A. L. (1984). Production of experimental endocarditis by coagulase-negative Staphylococci: variability in species virulence. *J. Infect. Dis.* 150 721–727. 10.1093/infdis/150.5.721 6491379

[B4] Baddour L. M. Wilson W. R. Bayer A. S. Fowler V. G. Bolger A. F. Levison M. E. (2005). Infective endocarditis: diagnosis, antimicrobial therapy, and management of complications: a statement for healthcare professionals from the Committee on Rheumatic Fever, Endocarditis, and Kawasaki Disease, Council on Cardiovascular Disease in the Young, and the Councils on Clinical Cardiology, Stroke, and Cardiovascular Surgery and Anesthesia, American Heart Association: endorsed by the Infectious Diseases Society of America. *Circulation* 111 e394–e434. 15956145 10.1161/CIRCULATIONAHA.105.165564

[B5] Balwit J. M. Langevelde P. V. Vann J. M. Proctor R. A. (1994). Gentamicin-resistant menadione and hemin auxotrophic *Staphylococcus aureus* persist within cultured endothelial cells. *J. Infect. Dis.* 170 1033–1037. 10.1093/infdis/170.4.1033 7930701

[B6] Bancsi M. J. Veltrop M. H. Bertina R. M. Thompson J. (1996). Role of phagocytosis in activation of the coagulation system in *Streptococcus sanguis* endocarditis. *Infect. Immun.* 64 5166–5170. 10.1128/iai.64.12.5166-5170.1996 8945561 PMC174503

[B7] Berube B. Wardenburg J. (2013). *Staphylococcus aureus* α-toxin: nearly a century of intrigue. *Toxins* 5 1140–1166. 10.3390/toxins5061140 23888516 PMC3717774

[B8] Bjerketorp J. Jacobsson K. Frykberg L. (2004). The von Willebrand factor-binding protein (vWbp) of *Staphylococcus aureus* is a coagulase. *FEMS Microbiol. Lett.* 234 309–314. 10.1111/j.1574-6968.2004.tb09549.x 15135538

[B9] Braden B. (1986). The surveyor’s area formula. *College Math. J.* 17 326–337.

[B10] Bussani R. De-Giorgio F. Pesel G. Zandonà L. Sinagra G. Grassi S. (2019). Overview and comparison of infectious endocarditis and non-infectious endocarditis: a review of 814 autoptic cases. *In Vivo* 33 1565–1572. 10.21873/invivo.11638 31471406 PMC6755013

[B11] Chavakis T. Hussain M. Kanse S. M. Peters G. Bretzel R. G. Flock J. I. (2002). *Staphylococcus aureus* extracellular adherence protein serves as anti-inflammatory factor by inhibiting the recruitment of host leukocytes. *Nat. Med.* 8 687–693. 10.1038/nm728 12091905

[B12] Claes J. Liesenborghs L. Peetermans M. Veloso T. R. Missiakas D. Schneewind O. (2017). Clumping factor A, von Willebrand factor-binding protein and von Willebrand factor anchor *Staphylococcus aureus* to the vessel wall. *J. Thromb. Haemost.* 15 1009–1019. 10.1111/jth.13653 28182324 PMC6232194

[B13] Dassy B. Hogan T. Foster T. J. Fournier J. M. (1993). Involvement of the accessory gene regulator (agr) in expression of type 5 capsular polysaccharide by *Staphylococcus aureus*. *J. Gen. Microbiol.* 139(Pt 6), 1301–1306. 10.1099/00221287-139-6-1301 8360622

[B14] De Meyer S. F. Deckmyn H. Vanhoorelbeke K. (2009). von Willebrand factor to the rescue. *Blood* 113 5049–5057. 10.1182/blood-2008-10-165621 19318682

[B15] Durack D. T. Beeson P. B. (1972). Experimental bacterial endocarditis. I. Colonization of a sterile vegetation. *Br. J. Exp. Pathol.* 53 44–49. 5014243 PMC2072378

[B16] Durack D. T. Beeson P. B. Petersdorf R. G. (1973). Experimental bacterial endocarditis. 3. Production and progress of the disease in rabbits. *Br. J. Exp. Pathol.* 54 142–151. 4700697 PMC2072580

[B17] Duthie E. S. Lorenz L. L. (1952). Staphylococcal coagulase; mode of action and antigenicity. *J. Gen. Microbiol.* 6 95–107. 14927856 10.1099/00221287-6-1-2-95

[B18] Else R. W. Holmes J. R. (1972). Cardiac pathology in the horse. 1. Gross pathology. *Equine Vet. J.* 4 1–8. 10.1111/j.2042-3306.1972.tb03868.x 4650883

[B19] Fraunholz M. Bernhardt J. Schuldes J. Daniel R. Hecker M. Sinha B. (2013). Complete genome sequence of *Staphylococcus aureus* 6850, a highly cytotoxic and clinically virulent methicillin-sensitive strain with distant relatedness to prototype strains. *Genome Announc.* 1:e00775-13. 10.1128/genomeA.00775-13 PMC3784790 24072870

[B20] Garrison P. K. Freedman L. R. (1970). Experimental endocarditis I. Staphylococcal endocarditis in rabbits resulting from placement of a polyethylene catheter in the right side of the heart. *Yale J. Biol. Med.* 42 394–410. 5431862 PMC2591670

[B21] Gibson G. W. Kreuser S. C. Riley J. M. Rosebury-Smith W. S. Courtney C. L. Juneau P. L. (2007). Development of a mouse model of induced *Staphylococcus aureus* infective endocarditis. *Comp. Med.* 57 563–569. 18246868

[B22] Giese B. Glowinski F. Paprotka K. Dittmann S. Steiner T. Sinha B. (2011). Expression of delta-toxin by *Staphylococcus aureus* mediates escape from phago-endosomes of human epithelial and endothelial cells in the presence of beta-toxin. *Cell. Microbiol.* 13 316–329. 10.1111/j.1462-5822.2010.01538.x 20946243

[B23] Goncharov N. V. Nadeev A. D. Jenkins R. O. Avdonin P. V. (2017). Markers and biomarkers of endothelium: when something is rotten in the state. *Oxid. Med. Cell. Longev.* 2017:9759735. 10.1155/2017/9759735 PMC5733214 29333215

[B24] Grosz M. Kolter J. Paprotka K. Winkler A.-C. Schäfer D. Chatterjee S. S. (2014). Cytoplasmic replication of *Staphylococcus aureus* upon phagosomal escape triggered by phenol-soluble modulin α. *Cell. Microbiol.* 16 451–465. 10.1111/cmi.12233 24164701 PMC3969633

[B25] Gupta R. K. Alba J. Xiong Y. Q. Bayer A. S. Lee C. Y. (2013). MgrA activates expression of capsule genes, but not the α-toxin gene in experimental *Staphylococcus aureus* endocarditis. *J. Infect. Dis.* 208 1841–1848. 10.1093/infdis/jit367 23901087 PMC3814835

[B26] Habib G. Lancellotti P. Antunes M. J. Bongiorni M. G. Casalta J.-P. Del Zotti F. (2015). 2015 ESC Guidelines for the management of infective endocarditis: the task force for the management of infective endocarditis of the European Society of Cardiology (ESC). Endorsed by: European Association for Cardio-Thoracic Surgery (EACTS), the European Association of Nuclear Medicine (EANM). *Eur. Heart J.* 36 3075–3128. 10.1093/eurheartj/ehv319 26320109

[B27] Hartleib J. Köhler N. Dickinson R. B. Chhatwal G. S. Sixma J. J. Hartford O. M. (2000). Protein A is the von Willebrand factor binding protein on *Staphylococcus aureus*. *Blood* 96 2149–2156. 10979960

[B28] Haslinger-Loffler B. Kahl B. C. Grundmeier M. Strangfeld K. Wagner B. Fischer U. (2005). Multiple virulence factors are required for *Staphylococcus aureus*-induced apoptosis in endothelial cells. *Cell. Microbiol.* 7 1087–1097. 10.1111/j.1462-5822.2005.00533.x 16008576

[B29] Haslinger-Loffler B. Wagner B. Bruck M. Strangfeld K. Grundmeier M. Fischer U. (2006). *Staphylococcus aureus* induces caspase-independent cell death in human peritoneal mesothelial cells. *Kidney Int.* 70 1089–1098. 10.1038/sj.ki.5001710 16871245

[B30] Héraïef E. Glauser M. P. Freedman L. R. (1982). Natural history of aortic valve endocarditis in rats. *Infect. Immun.* 37 127–131. 10.1128/iai.37.1.127-131.1982 7049946 PMC347499

[B31] Highman B. Altland P. D. Roshe J. (1959). Staphylococcal endocarditis and glomerulonephritis in dogs. *Circ. Res.* 7 982–987. 10.1161/01.res.7.6.982 14401760

[B32] Highman B. Roshe J. Altland P. D. (1956). Production of endocarditis with *Staphylococcus aureus* and *Streptococcus mitis* in dogs with aortic insufficiency. *Circ. Res.* 4 250–256. 10.1161/01.res.4.3.250 13317014

[B33] Hoen B. Duval X. (2013). Clinical practice. Infective endocarditis. *N. Engl. J. Med.* 368 1425–1433. 23574121 10.1056/NEJMcp1206782

[B34] Hoerr V. Franz M. Pletz M. W. Diab M. Niemann S. Faber C. (2018). *S. aureus* endocarditis: clinical aspects and experimental approaches. *Int. J. Med. Microbiol.* 308 640–652. 10.1016/j.ijmm.2018.02.004 29526448

[B35] Höök M. Foster T. J. (2000). “Molecular basis of adherence of *staphylococcus aureus* to biomaterials,” in *Infections Associated with Indwelling Medical Devices*, eds Waldvogel F. A. Bisno A. L. (Washington, D.C: ASM Press), 27–39. 10.1128/9781555818067.ch2

[B36] Johnson C. M. Bahn R. C. Fass D. N. (1986). Experimental porcine infective endocarditis: description of a clinical model. *Vet. Pathol.* 23 780–782. 10.1177/030098588602300620 3811145

[B37] Johnson M. Cockayne A. Morrissey J. A. (2008). Iron-regulated biofilm formation in *Staphylococcus aureus* Newman requires ica and the secreted protein Emp. *Infect. Immun.* 76 1756–1765. 10.1128/iai.01635-07 18268030 PMC2292859

[B38] Jones J. E. (1969). The experimental production of streptococcal endocarditis in the pig. *J. Pathol.* 99 307–318. 10.1002/path.1710990406 5401242

[B39] Jones J. E. T. (1982). Experimental streptococcal endocarditis in the pig: the development of lesions 18 to 48 hours after inoculation. *J. Comp. Pathol.* 92 301–308. 10.1016/0021-9975(82)90089-5 7085946

[B40] Joost I. Blass D. Burian M. Goerke C. Wolz C. von Müller L. (2009). Transcription analysis of the extracellular adherence protein from *Staphylococcus aureus* in authentic human infection and *in vitro*. *J. Infect. Dis.* 199 1471–1478. 10.1086/598484 19351261

[B41] Juuti K. M. Sinha B. Werbick C. Peters G. Kuusela P. I. (2004). Reduced adherence and host cell invasion by methicillin-resistant *Staphylococcus aureus* expressing the surface protein Pls. *J. Infect. Dis.* 189 1574–1584. 10.1086/383348 15116292

[B42] Kalinka J. Hachmeister M. Geraci J. Sordelli D. Hansen U. Niemann S. (2014). *Staphylococcus aureus* isolates from chronic osteomyelitis are characterized by high host cell invasion and intracellular adaptation, but still induce inflammation. *Int. J. Med. Microbiol.* 304 1038–1049. 10.1016/j.ijmm.2014.07.013 25129555

[B43] Kazlouskaya V. Malhotra S. Lambe J. Idriss M. H. Elston D. Andres C. (2013). The utility of elastic Verhoeff-Van Gieson staining in dermatopathology. *J. Cutan. Pathol.* 40 211–225. 10.1111/cup.12036 23216221

[B44] Kobayashi S. D. Malachowa N. DeLeo F. R. (2015). Pathogenesis of *Staphylococcus aureus* abscesses. *Am. J. Pathol.* 185 1518–1527. 10.1016/j.ajpath.2014.11.030 25749135 PMC4450319

[B45] Kolar S. L. Liu G. Y. (2016). Targeting bacterial abscess formation. *EBioMedicine* 12 16–17. 10.1016/j.ebiom.2016.10.017 27765641 PMC5078668

[B46] Kreitmann L. Montaigne D. Launay D. Morell-Dubois S. Maillard H. Lambert M. (2020). Clinical characteristics and outcome of patients with infective endocarditis diagnosed in a department of internal medicine. *J. Clin. Med.* 9:864. 10.3390/jcm9030864 32245196 PMC7141516

[B47] Lam T.-T. Giese B. Chikkaballi D. Kuhn A. Wolber W. Pane-Farre J. (2010). Phagolysosomal integrity is generally maintained after *Staphylococcus aureus* invasion of nonprofessional phagocytes but is modulated by strain 6850. *Infect. Immun.* 78 3392–3403. 10.1128/iai.00012-10 20530231 PMC2916288

[B48] Li X. Wang X. Thompson C. D. Park S. Park W. B. Lee J. C. (2016). Preclinical efficacy of clumping factor A in prevention of *Staphylococcus aureus* infection. *mBio* 7:e02232-15. 10.1128/mBio.02232-15 PMC4742718 26838725

[B49] Liesenborghs L. Meyers S. Lox M. Criel M. Claes J. Peetermans M. (2019). *Staphylococcus aureus* endocarditis: distinct mechanisms of bacterial adhesion to damaged and inflamed heart valves. *Eur. Heart J.* 40 3248–3259. 10.1093/eurheartj/ehz175 30945735 PMC7963134

[B50] Liesenborghs L. Verhamme P. Vanassche T. (2018). *Staphylococcus aureus*, master manipulator of the human hemostatic system. *J. Thromb. Haemost.* 16 441–454. 10.1111/jth.13928 29251820

[B51] Mainiero M. Goerke C. Geiger T. Gonser C. Herbert S. Wolz C. (2010). Differential target gene activation by the *Staphylococcus aureus* two-component system saeRS. *J. Bacteriol.* 192 613–623. 10.1128/jb.01242-09 19933357 PMC2812464

[B52] Maira-Litrán T. Kropec A. Abeygunawardana C. Joyce J. Mark G. Goldmann D. A. (2002). Immunochemical properties of the staphylococcal poly-N-acetylglucosamine surface polysaccharide. *Infect. Immun.* 70 4433–4440. 10.1128/iai.70.8.4433-4440.2002 12117954 PMC128161

[B53] Manandhar S. Singh A. Varma A. Pandey S. Shrivastava N. (2018). Evaluation of methods to detect *in vitro* biofilm formation by staphylococcal clinical isolates. *BMC Res. Notes* 11:714. 10.1186/s13104-018-3820-9 30305150 PMC6180658

[B54] McDevitt D. Foster T. J. (1995). Variation in the size of the repeat region of the fibrinogen receptor (clumping factor) of *Staphylococcus aureus* strains. *Microbiology* 141(Pt 4), 937–943. 10.1099/13500872-141-4-937 7773396

[B55] Mehta R. Athar M. Girgis S. Hassan A. Becker R. C. (2019). Acquired Von Willebrand Syndrome (AVWS) in cardiovascular disease: a state of the art review for clinicians. *J. Thromb. Thrombolysis* 48 14–26. 10.1007/s11239-019-01849-2 31004311 PMC6624837

[B56] Moreillon P. Que Y. A. Bayer A. S. (2002). Pathogenesis of streptococcal and staphylococcal endocarditis. *Infect. Dis. Clin. North Am.* 16 297–318. 10.1016/s0891-5520(01)00009-5 12092474

[B57] Novick R. P. (2003). Autoinduction and signal transduction in the regulation of staphylococcal virulence. *Mol. Microbiol.* 48 1429–1449. 10.1046/j.1365-2958.2003.03526.x 12791129

[B58] O’Grady N. P. Alexander M. Dellinger E. P. Gerberding J. L. Heard S. O. Maki D. G. (2002). Guidelines for the prevention of intravascular catheter–related infections. *Clin. Infect. Dis* 35 1281–1307. 10.1086/502007 12517020

[B59] Pant J. Goudie M. J. Chaji S. M. Johnson B. W. Handa H. (2018). Nitric oxide releasing vascular catheters for eradicating bacterial infection. *J. Biomed. Mater. Res. B Appl. Biomater.* 106 2849–2857. 10.1002/jbm.b.34065 29266734 PMC6013312

[B60] Pappelbaum K. I. Gorzelanny C. Grässle S. Suckau J. Laschke M. W. Bischoff M. (2013). Ultralarge von Willebrand factor fibers mediate luminal *Staphylococcus aureus* adhesion to an intact endothelial cell layer under shear stress. *Circulation* 128 50–59. 10.1161/circulationaha.113.002008 23720451

[B61] Ring J. Hoerr V. Tuchscherr L. Kuhlmann M. T. Löffler B. Faber C. (2014). MRI visualization of *Staphyloccocus aureus*-induced infective endocarditis in mice. *PLoS One* 9:e107179. 10.1371/journal.pone.0107179 25229324 PMC4167704

[B62] Roberts J. C. Krueger R. L. Peak K. K. Veguilla W. Cannons A. C. Amuso P. T. (2006). Community-associated methicillin-resistant *Staphylococcus aureus* epidemic clone USA300 in isolates from Florida and Washington. *J. Clin. Microbiol.* 44 225–226. 10.1128/jcm.44.1.225-226.2006 16390975 PMC1351968

[B63] Rowlands D. T. Vakilzadeh J. Sherwood B. F. LeMay J. C. (1970). Experimental bacterial endocarditis in the opossum (*Didelphis virginiana*). I. Valvular changes following a single injection of bacteria in unmodified adult opossums. *Am. J. Pathol.* 58 295–304. 5437267 PMC2032822

[B64] Sadler J. E. (1998). Biochemistry and genetics of von Willebrand factor. *Annu. Rev. Biochem.* 67 395–424. 10.1146/annurev.biochem.67.1.395 9759493

[B65] Sande M. A. Zak O. (1999). *Handbook of Animal Models of Infection: Experimental Models in Antimicrobial Chemotherapy.* San Diego, CA: Academic Press.

[B66] Sause W. E. Copin R. O’malley A. Chan R. Morrow B. J. Buckley P. T. (2017). *Staphylococcus aureus* strain Newman D2C contains mutations in major regulatory pathways that cripple its pathogenesis. *J. Bacteriol.* 199:e00476-17. 10.1128/JB.00476-17 PMC5686614 28924032

[B67] Seidl K. Bayer A. S. McKinnell J. A. Ellison S. Filler S. G. Xiong Y. Q. (2011). *In vitro* endothelial cell damage is positively correlated with enhanced virulence and poor vancomycin responsiveness in experimental endocarditis due to methicillin-resistant *Staphylococcus aureus*. *Cell. Microbiol.* 13 1530–1541. 10.1111/j.1462-5822.2011.01639.x 21777408 PMC3173605

[B68] Seidl K. Zinkernagel A. S. (2013). The MTT assay is a rapid and reliable quantitative method to assess *Staphylococcus aureus* induced endothelial cell damage. *J. Microbiol. Methods* 92 307–309. 10.1016/j.mimet.2012.12.018 23275136

[B69] Shrum B. Anantha R. V. Xu S. X. Donnelly M. Haeryfar S. M. M. McCormick J. K. (2014). A robust scoring system to evaluate sepsis severity in an animal model. *BMC Res. Notes* 7:233. 10.1186/1756-0500-7-233 24725742 PMC4022086

[B70] Singer M. Deutschman C. S. Seymour C. W. Shankar-Hari M. Annane D. Bauer M. (2016). The third international consensus definitions for sepsis and septic shock (Sepsis-3). *JAMA* 315 801–810. 26903338 10.1001/jama.2016.0287 PMC4968574

[B71] Starke R. D. Ferraro F. Paschalaki K. E. Dryden N. H. McKinnon T. A. J. Sutton R. E. (2011). Endothelial von Willebrand factor regulates angiogenesis. *Blood* 117 1071–1080. 21048155 10.1182/blood-2010-01-264507 PMC3035068

[B72] Strobel M. Pförtner H. Tuchscherr L. Völker U. Schmidt F. Kramko N. (2016). Post-invasion events after infection with *Staphylococcus aureus* are strongly dependent on both the host cell type and the infecting *S. aureus* strain. *Clin. Microbiol. Infect.* 22 799–809. 10.1016/j.cmi.2016.06.020 27393124

[B73] Subedi S. Jennings Z. Chen S. C.-A. (2017). Laboratory approach to the diagnosis of culture-negative infective endocarditis. *Heart Lung Circ.* 26 763–771. 10.1016/j.hlc.2017.02.009 28372886

[B74] Surewaard B. G. J. Thanabalasuriar A. Zeng Z. Tkaczyk C. Cohen T. S. Bardoel B. W. (2018). α-toxin induces platelet aggregation and liver injury during *Staphylococcus aureus* sepsis. *Cell Host Microbe* 24 271–284.e3. 10.1016/j.chom.2018.06.017 30033122 PMC6295203

[B75] Traber K. E. Lee E. Benson S. Corrigan R. Cantera M. Shopsin B. (2008). *agr* function in clinical *Staphylococcus aureus* isolates. *Microbiology* 154 2265–2274. 18667559 10.1099/mic.0.2007/011874-0 PMC4904715

[B76] Tuchscherr L. Medina E. Hussain M. Völker W. Heitmann V. Niemann S. (2011). *Staphylococcus aureus* phenotype switching: an effective bacterial strategy to escape host immune response and establish a chronic infection. *EMBO Mol. Med.* 3 129–141. 10.1002/emmm.201000115 21268281 PMC3395110

[B77] Vandenesch F. Lina G. Henry T. (2012). *Staphylococcus aureus* hemolysins, bi-component leukocidins, and cytolytic peptides: a redundant arsenal of membrane-damaging virulence factors? *Front. Cell. Infect. Microbiol.* 2:12. 10.3389/fcimb.2012.00012 22919604 PMC3417661

[B78] Vann J. M. Proctor R. A. (1987). Ingestion of *Staphylococcus aureus* by bovine endothelial cells results in time- and inoculum-dependent damage to endothelial cell monolayers. *Infect. Immun.* 55 2155–2163. 10.1128/iai.55.9.2155-2163.1987 3623696 PMC260672

[B79] Veloso T. R. Amiguet M. Rousson V. Giddey M. Vouillamoz J. Moreillon P. (2011). Induction of experimental endocarditis by continuous low-grade bacteremia mimicking spontaneous bacteremia in humans. *Infect. Immun.* 79 2006–2011. 10.1128/iai.01208-10 21321073 PMC3088130

[B80] von Eiff C. Heilmann C. Peters G. (1999). New aspects in the molecular basis of polymer-associated infections due to staphylococci. *Eur. J. Clin. Microbiol. Infect. Dis.* 18 843–846. 10.1007/s100960050417 10691193

[B81] Werdan K. Dietz S. Loffler B. Niemann S. Bushnaq H. Silber R. E. (2014). Mechanisms of infective endocarditis: pathogen-host interaction and risk states. *Nat. Rev. Cardiol.* 11 35–50. 10.1038/nrcardio.2013.174 24247105

[B82] Xie Y. Tu B. Xu Z. Zhang X. Bi J. Zhao M. (2017). Bacterial distributions and prognosis of bloodstream infections in patients with liver cirrhosis. *Sci. Rep.* 7:11482. 10.1038/s41598-017-11587-1 PMC5597589 28904387

[B83] Zimmerli W. Waldvogel F. A. Vaudaux P. Nydegger U. E. (1982). Pathogenesis of foreign body infection: description and characteristics of an animal model. *J. Infect. Dis.* 146 487–497. 10.1093/infdis/146.4.487 7119479

