# Supplementary figures and images for: Isolating Crucial Steps in Induction of Infective Endocarditis With Preclinical Modeling of Host Pathogen Interaction
Source: Front Microbiol. 2020 Jun 18;11:1325. doi: 10.3389/fmicb.2020.01325 (PMC7314968; doi:10.3389/fmicb.2020.01325)

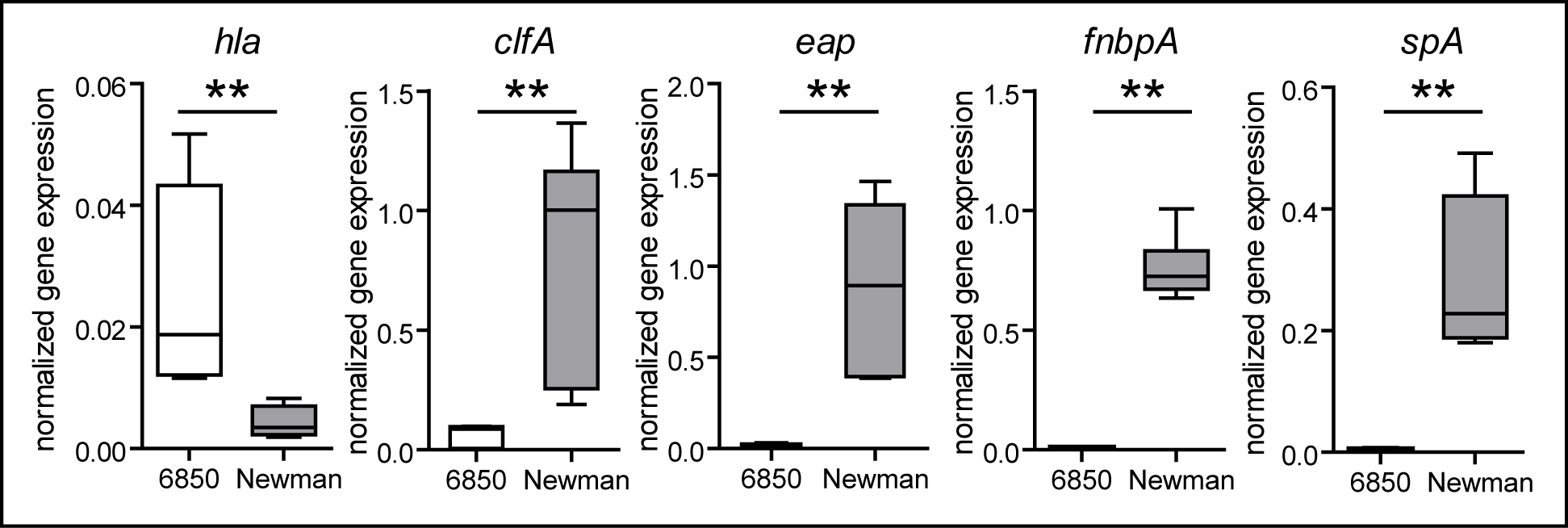

Supplement: FIGURE S1 — Gene-expression of the α toxin (hla) and different adhesion molecules such as staphylococcal protein A (spA), extracellular adherence protein (eap), Clumping factor A (clfA) and Fibronectin binding protein A (fnbA) in S. aureus 6850 (white boxes) and Newman (gray boxes). The results (n = 6 out of three independent cultures) are displayed as box and whiskers plots, with data between first and third quartiles, the band in the box stands for the second quartile (=median). Whiskers represent lowest and highest data within 1.5 interquartile ranges of the lower and upper quartile. ∗p < 0.05, ∗∗p < 0.01. [file Image_1.JPEG]

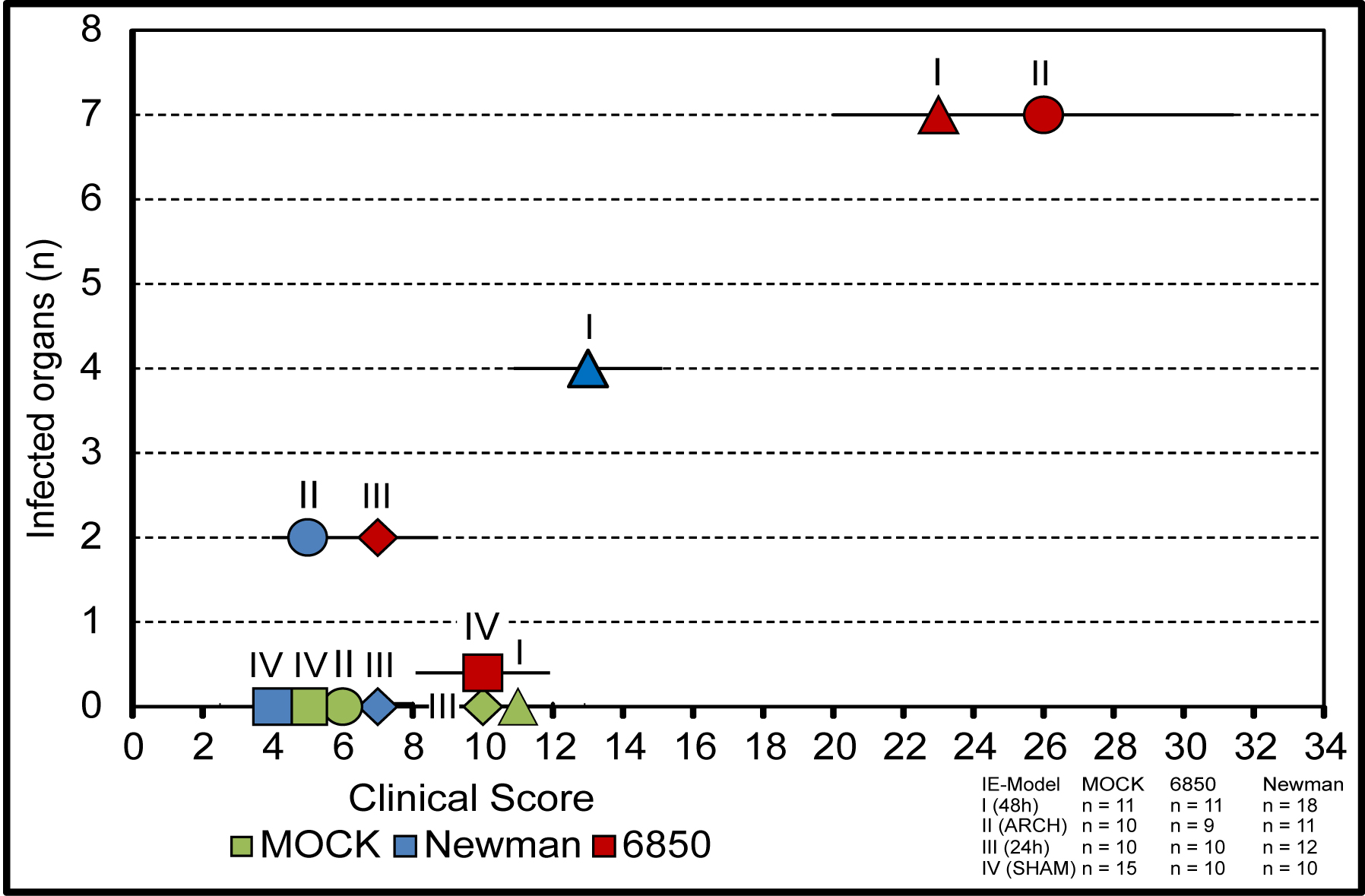

Supplement: FIGURE S2 — Correlation of number of infected organs and clinical score, in all four models of IE induced with either S. aureus 6850 or Newman. Mean clinical score (±SEM) of each experimental group at 24 h post infection and the corresponding number of infected organs (≥105 CFU / mg tissue, see Figure 4) is shown. [file Image_2.JPEG]
