# Supplementary material for: Isolating Crucial Steps in Induction of Infective Endocarditis With Preclinical Modeling of Host Pathogen Interaction
Source: Front Microbiol. 2020 Jun 18;11:1325. doi: 10.3389/fmicb.2020.01325 (PMC7314968; doi:10.3389/fmicb.2020.01325)
Supplement: TABLE S1 — Clinical Score. [file Table_1.docx]

Supplementary table 1

**Clinical Score**

Table 1: To assess severity of the disease in the experimental course the following clinical score was used.

| **Variable** | **Score and description** |
| --- | --- |
| Weight [g] | 0 - no changes or weight gain |
|  | 1 - weight loss < 5 % |
|  | 5 - weight loss 5 – 10 % |
|  | 10 - weight loss 11 – 19 % |
|  | 20 - weight loss ≥ 20 % |
| Temperature [°C] | 1 - body temperature rise up to 1 °C |
|  | 5 - body temperature rise up to 2 °C |
|  | 10 - body temperature rise up to 3 °C |
|  | 20 - body temperature rise up to 4 °C |
| Respiration quality | 0 - normal |
|  | 10 - changed breathing (fast or slow) |
|  | 20 - laboured breathing |
| Appearance | 0 - coat smooth, shiny, body orifices clean, eyes clear |
|  | 1 - decreased body care, temporary eye or nose discharge, hair loss |
|  | 5 - coat dull, eyes cloudy or sticky, muscle tone increased, body orifices unkempt |
|  | 10 - uncultivated, shaggy coat, body orifices: sticky and damp, bloody faeces, altered posture, prolapse, constant eye or nose discharge, reduced grip strength, bite wounds |
|  | 20 - uncultivated, shaggy or dirty coat, dehydration, grip strength not present, stilty gait, tremors, cramps, paralysis |
| Behaviour | 0 - animal shows normal behaviour again a few hours after surgery (locomotion, curiosity, social contact, sleeping, reaction to touching) |
|  | 1 - animal sleeps more often in the first hours after surgery, but the mouse reacts immediately when touched |
|  | 5 - motor activity conspicuously restricted or increased |
|  | 10 - lethargy, behavioral stereotypes, pronounced hyperkinetics, coordination disorders, pain reaction at touch |
|  | 20 - self-amputation |
| Assessment of wound healing after surgery | 0 - surgical wound dry, wound healing unobtrusive |
|  | 10 - OP wound swollen, sticky or moist |
|  | 20 - significant suppurative foci in the area of the surgical wound |
|  |  |
| Evaluation | Sum of all scores - actions |
| Load factor 0 = no load | 0 - none |
| Load factor 1 = low load | 1-9 - careful observation |
| Load factor 2 = moderate load | 10 -19 - analgesia on a maximum of three days post op; if necessary wound care; intensive observation; consultation with the responsible scientist and the responsible veterinarian |
| Load factor 3 = high load | 20 or more - high grade load euthanasia |
