# Supplementary material for: Isolating Crucial Steps in Induction of Infective Endocarditis With Preclinical Modeling of Host Pathogen Interaction
Source: Front Microbiol. 2020 Jun 18;11:1325. doi: 10.3389/fmicb.2020.01325 (PMC7314968; doi:10.3389/fmicb.2020.01325)
Supplement: TABLE S2 — Antibiotype of Staphylococcus aureus strains 6850 and Newman. [file Table_2.DOCX]

Supplementary table 2

**Antibiotype of *Staphylococcus aureus* strains 6850 and Newman**

Table 2: To assess antibiotic sensitivity and resistance to both studied *S. aureus* strains.

| Antibiotype of *Staphylococcus aureus* 6850 |
| --- |

| Antibiotic | MIC [µg/ml] | Inter-pretation | Antbiotic | MIC [µg/ml] | Inter-  pretation |
| --- | --- | --- | --- | --- | --- |
| Cefoxitin-Screen | NEG |  | Levofloxacin | <=0.12 | I |
| Benzylpenicillin | >= 0.5 | R | Inducible Clindamycin  resistance | NEG | - |
| + Amoxicillin |  | R | + Azithromycin |  | S |
| + Ampicillin |  | R | + Clarithromycin |  | S |
| + Amoxicillin/Clavulansäure |  | S | Erythromycin | 1 | S |
| + Ampicillin/Sulbactam |  | S | Clindamycin | 0.25 | S |
| + Piperacillin |  | R | Linezolid | 2 | S |
| + Piperacillin/Tazobactam |  | S | Daptomycin | 0.25 | S |
| Oxacillin | <=0.25 | S | Teicoplanin | <=0.5 | S |
| + Cefaclor |  | S | Vancomycin | <=0.5 | S |
| + Cefazolin |  | S | Tetracyclin | <=1 | S |
| + Cefuroxim |  | S | Tigecycline | <=0.12 | S |
| + Ceftriaxon |  | S | Fosfomycin | <=8 | S |
| + Ertapenem |  | S | Fusidinsäure | <=0.5 | S |
| + Imipenem |  | S | Mupirocin | <=1 | S |
| + Meropenem |  | S | Rifampicin | <=0.03 | S |
| Gentamicin | <=0.5 | S | Trimethoprim/Sulfamethoxazol | <=10 | S |
| MIC = Minimal inhibitory concentration; R = resistant; S = sensitive; NEG = negative; I = intermediate :  moderately sensitive/intermediately sensitive | | | | | |

| Antibiotype of *Staphylococcus aureus* Newman |
| --- |

| Antibiotic | MIC [µg/ml] | Inter  pretation | Antibiotic | MIC [µg/ml] | Inter-  pretation |
| --- | --- | --- | --- | --- | --- |
| Cefoxitin-Screen | NEG |  | Levofloxacin | <=0.12 | I |
| Benzylpenicillin | <= 0.03 | S | Inducible Clindamycin  resistance | NEG | - |
| + Amoxicillin |  | S | + Azithromycin |  | S |
| + Ampicillin |  | S | + Clarithromycin |  | S |
| + Amoxicillin/Clavulansäure |  | S | Erythromycin | 0.5 | S |
| + Ampicillin/Sulbactam |  | S | Clindamycin | 0.25 | S |
| + Piperacillin |  | S | Linezolid | 2 | S |
| + Piperacillin/Tazobactam |  | S | Daptomycin | 0.5 | S |
| Oxacillin | <=0.25 | S | Teicoplanin | 2 | S |
| + Cefaclor |  | S | Vancomycin | 1 | S |
| + Cefazolin |  | S | Tetracyclin | <=1 | S |
| + Cefuroxim |  | S | Tigecycline | <=0.12 | S |
| + Ceftriaxon |  | S | Fosfomycin | <=8 | S |
| + Ertapenem |  | S | Fusidinsäure | <=0.5 | S |
| + Imipenem |  | S | Mupirocin | <=1 | S |
| + Meropenem |  | S | Rifampicin | <=0.03 | S |
| Gentamicin | <=0.5 | S | Trimethoprim/Sulfamethoxazol | <=10 | S |
| MIC = Minimal inhibitory concentration; R = resistant; S = sensitive; NEG = negative; I = intermediate :  moderately sensitive/intermediately sensitive | | | | | |
